# Reactivation of *Cytomegalovirus* in Colonic Tuberculosis in an Immunocompetent Patient

**DOI:** 10.1155/crdi/5455768

**Published:** 2026-09-08

**Authors:** Aprilgate Wang, Samuel Ji, Sarah Semon, Sarah Waldman, Minh-Vu H. Nguyen

**Affiliations:** ^1^ Department of Internal Medicine, University of California-Davis, UC Davis Health, Sacramento, USA; ^2^ Department of Internal Medicine, Division of Gastroenterology and Hepatology, University of California-Davis, UC Davis Health, Sacramento, USA; ^3^ Department of Internal Medicine, Division of Infectious Disease, University of California-Davis, UC Davis Health, Sacramento, USA

**Keywords:** CMV reactivation, gastrointestinal tuberculosis, terminal ileitis

## Abstract

*Cytomegalovirus* (CMV) reactivation in *Mycobacterium tuberculosis* (TB) infection is rare and has been only reported in immunocompromised patients. In this case report, we describe a patient with Type 2 diabetes with acute on chronic abdominal pain found to have miliary TB with terminal ileitis and ascending colon ulcers with biopsy‐positive TB and CMV. The decision was made not to treat the local CMV reactivation, and he improved solely on standard drug‐susceptible antituberculous therapy. This is the first reported case of gastrointestinal TB and CMV coinfection in a patient without known classic immunodeficiency.

## 1. Introduction

Gastrointestinal (GI) TB is an uncommon presentation of extrapulmonary tuberculosis and accounts for 6%–13% of extrapulmonary manifestations [1]. Due to its nonspecific presentation, it resembles inherently similar and more common pathologies such as ulcers, malignancy, appendicitis, colitis, spontaneous bacterial peritonitis, and inflammatory bowel disease. For example, differentiating GI TB and Crohn’s disease is often difficult. Both are chronic inflammatory granulomatous diseases that affect the terminal ileum and require multiple positive diagnostic markers for a definitive diagnosis [2]. Both are considered immunocompromised states that can lead to reactivation of CMV.

CMV infection is very common, and most infected humans experience lifelong latency [3]. CMV reactivation leading to clinical disease is predominantly encountered in immunocompromised hosts. These hosts have impaired immune systems, congenital or acquired, that make then susceptible to otherwise ordinarily benign conditions. There is increased interest in examining acute critical illness as a state associated with CMV reactivation [4]. Reactivation is known to cause significant burden of disease in major organ systems. In patients with TB, evidence of CMV reactivation and a relationship between the two pathogens has been established although the exact nature remains unclear. Diffuse lower gastrointestinal bleed in GI TB should raise suspicion for CMV colitis, especially in immunocompromised patients.

## 2. Case Presentation

An 81‐year‐old man born in Vietnam who immigrated to the United States 20 years ago with a past medical history significant for Type 2 diabetes presented to the emergency department with a one‐year history of chronic progressive abdominal pain that worsened in the past three months. The abdominal pain was dull, aching, localized to the epigastrium, and exacerbated with eating. He reported unintentional weight loss of 20 lbs during this time. For the past 3 days, he has had fevers and chills. He denied diarrhea, constipation, nausea, cough, and night sweats. He had an esophagogastroduodenoscopy outpatient and was treated for gastritis and *H. pylori* without improvement of symptoms. He had visited Vietnam frequently since immigration, and he was there most recently for 2 weeks and returned 5 days prior to presentation.

**FIGURE 1:**
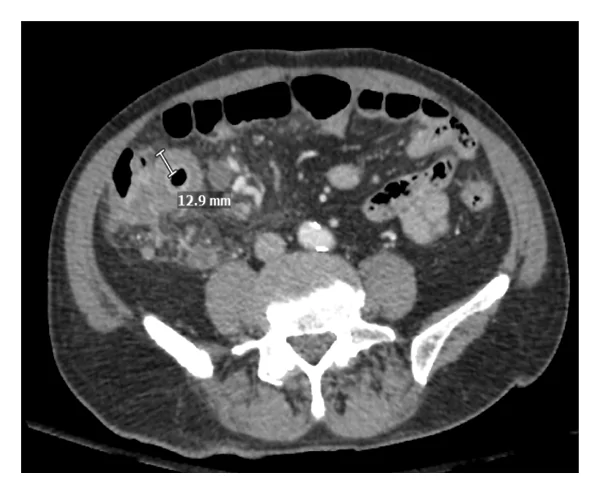
Thickening of the terminal ileum measuring 12.9 mm seen on CT of the abdomen and pelvis with contrast consistent with terminal ileitis.

On admission, vital signs were significant for a temperature of 39.2°C. Physical examination was notable for a soft, distended abdomen with epigastric tenderness on palpation. Complete blood count and chemistries were unremarkable. He had elevated inflammatory makers including erythrocyte sedimentation rate (126 mm/hr; reference range 0–20 mm/hr), C‐reactive protein (28 mg/dL; reference range < 0.5 mg/dL), and ferritin (917 ng/mL; reference range 30–400 ng/mL). Chest radiograph showed no acute cardiopulmonary process. HIV antibody and syphilis antibody tests were nonreactive. The patient had a positive interferon‐gamma release assay (QuantiFERON‐TB GOLD Plus, QIAGEN, Hilden, Germany) in November 2023. Computed tomography (CT) scan of the abdomen and pelvis with contrast revealed multiple necrotic gastrohepatic and periesophageal lymph nodes and terminal ileitis (Figure 1).

**FIGURE 2:**
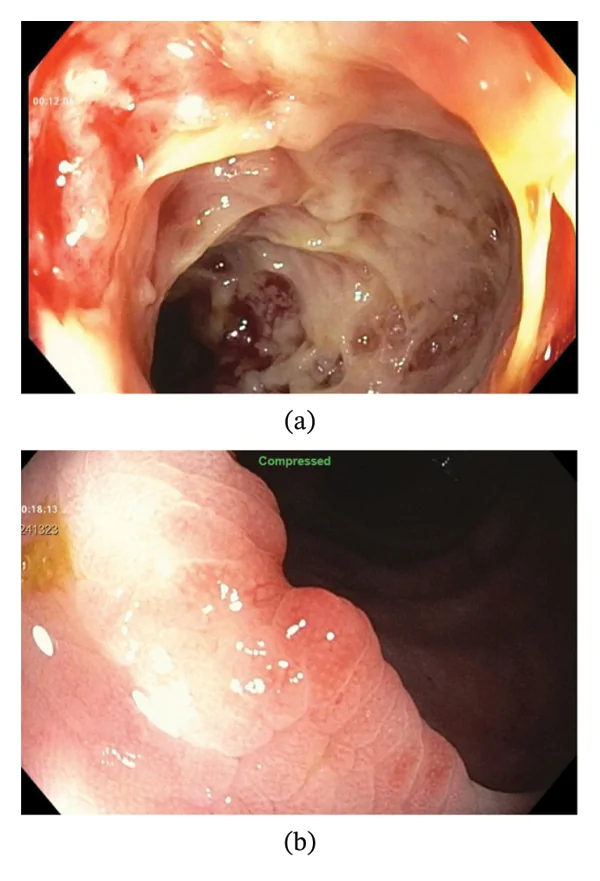
(a) Colonoscopy images showing the narrowed, friable, scarred ileum. (b) Ulcer of the ascending colon.

**FIGURE 3:**
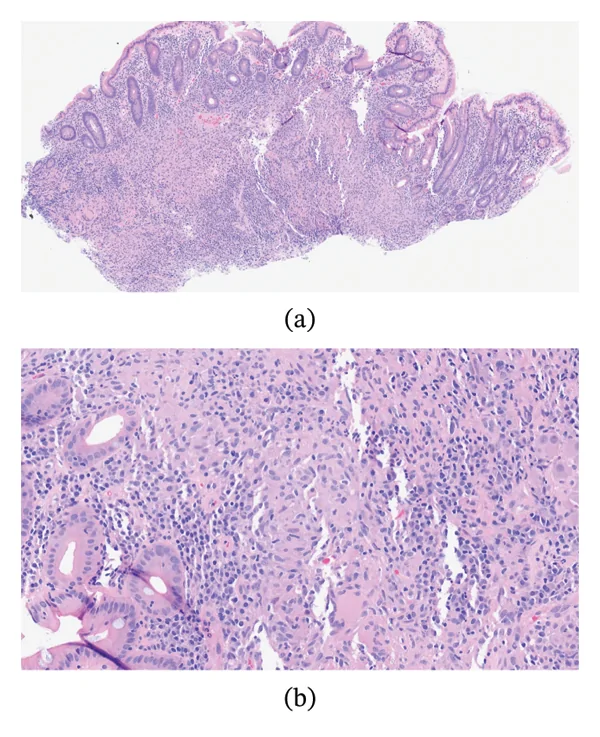
(a) Low power H&E stain of the ascending colon showing necrotizing granulomatous inflammation. (b) High power H&E stain of the ascending colon showing necrotizing granulomatous inflammation.

The patient continued to have fevers despite empiric therapy with ceftriaxone for presumed intraabdominal infection. A colonoscopy was performed and showed severely edematous, erythematous, friable, and scarred mucosa with loss of vascular pattern and pallor in the terminal ileum (Figure 2a) and narrowed ileocecal valve. Biopsies were taken, including an ulcer of the ascending colon (Figure 2b). Initial hematoxylin and eosin (H&E) stain of the ulcer of the ascending colon showed necrotizing granulomatous inflammation (Figure 3a,b). Infectious Diseases (ID) Consultation service was asked to evaluate the patient due to high suspicion of TB.

**FIGURE 4:**
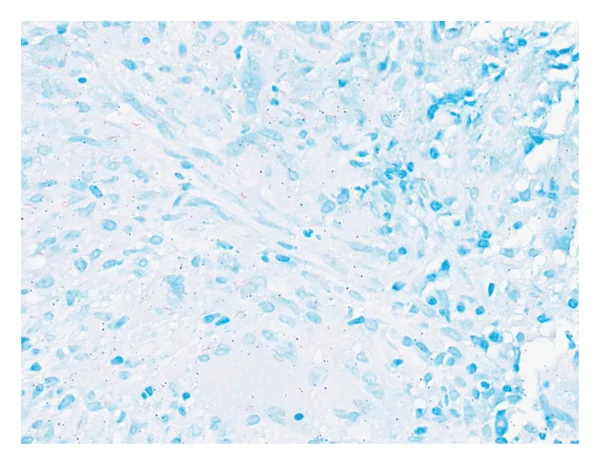
Acid‐fast stain of the ascending colon showing rod‐shaped organisms consistent with *Mycobacterium*.

**FIGURE 5:**
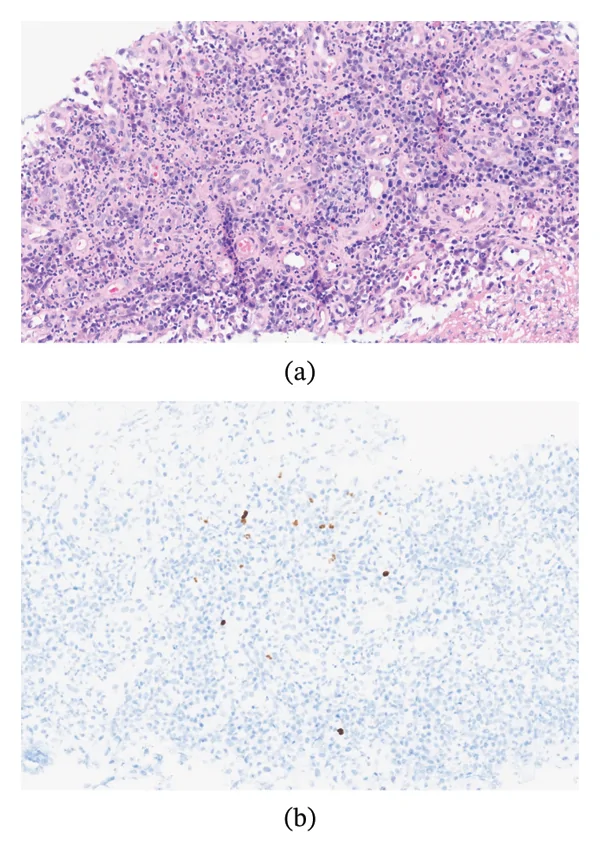
(a) H&E stain of the terminal ileum showing ulceration and granulation tissue. (b) Immunohistochemistry stain of the terminal ileum with scattered viral inclusions consistent with *Cytomegalovirus*.

Acid‐fast staining showed rod‐shaped organisms consistent with *Mycobacterium* (Figure 4). Mycobacterial urine and stool studies were positive for acid‐fast bacilli. Xpert MTB/RIF assay (Cepheid, Sunnyvale, CA, USA) on the urine specimen was positive for *Mycobacterium tuberculosis* without rifampin resistance. CT of the chest revealed miliary nodules consistent with miliary tuberculosis. CT of the head and neck revealed numerous cervical and supraclavicular necrotic lymphadenopathy. H&E stain of the terminal ileum showed ulceration and granulation tissue (Figure 5a), and immunohistochemistry was consistent with CMV infection (Figure 5b). CMV viral load was 61 U/mL (cobas CMV test, Roche 6800, Roche Diagnostics, no reference range available). The patient was started on antituberculosis therapy of isoniazid 300 mg daily with pyridoxine 50 mg daily, rifampin 600 mg daily, ethambutol 800 mg daily, and pyrazinamide 1000 mg daily with monthly monitoring of complete blood count and chemistries for drug toxicity. The decision was made not to treat the concomitant CMV reactivation with the prediction that the local CMV reactivation was driven by TB and would resolve with adequate treatment of tuberculosis.

The patient was discharged to follow up outpatient for Directly Observed Therapy (DOT) at his local tuberculosis clinic. Phenotypic drug susceptibility testing confirmed that his MTB isolate was susceptible to all first‐line antituberculosis drugs. Following completion of 6 months of antituberculosis therapy, his symptoms resolved. A repeat CT of the abdomen and pelvis with contrast at that time showed decreased terminal ileal wall thickening with resolution of surrounding inflammatory stranding and lymphadenopathy.

## 3. Discussion

This is the first published case of tuberculous ileitis with simultaneous gastrointestinal CMV disease in an immunocompetent patient. CMV reactivation and gastrointestinal tuberculous coinfection has been described in only four other case studies, all in immunocompromised patients. The first was in 2013 in a 32‐year‐old man with acquired immunodeficiency syndrome (AIDS) who presented with hemorrhagic shock from lower gastrointestinal bleeding that required surgical intervention [5]. The second was in a 41‐year‐old man with AIDS who reported profuse hematochezia and epigastric burning found to have esophageal and colon ulcers [6]. The last two, both published in 2022, were in renal transplant patients on immunosuppressive therapy. The first presented with hematochezia and was found to have a bleeding ulcer in the terminal ileum [7], and the second presented with chronic diarrhea with colitis and stenosis of the ascending lumen [8]. All cases reported histopathology showing owl eye inclusion bodies or a detectable CMV viral load.

Tuberculosis and CMV reactivation disease have been shown to be connected. CMV coinfection in tuberculosis is more commonly studied in children, and studies focus on possible immunological drivers of CMV that predispose them to active TB [9–11]. The actual pathophysiology of their interaction is not yet understood, but CMV coinfection in tuberculosis is associated with worse outcomes [10]. There is also emerging evidence that TB in turn affects CMV pathogenesis, thus suggesting a reciprocal relationship. Increased CMV antibodies and interferon‐γ response were associated with active pulmonary TB cases. TB cases were three times more likely to be infected with CMV, and more severe TB cases had greater levels of CMV antibodies compared to mild TB cases [11]. Separate from TB, CMV reactivation has also been studied in critically ill patients. In these patients, admitted for a spectrum of pathologies including bacterial sepsis, trauma, burn, major surgeries, and cardiac events, CMV reactivation has shown to be associated with worse clinical outcomes [12, 13]. One study [14] carried out in immunocompetent patients on mechanical ventilation showed patients with CMV reactivation had significantly worse outcomes including longer hospitalizations, prolonged intubation, and increased mortality. There is no clear evidence if a relationship exists between CMV reactivation and Type 2 diabetes.

The patient’s only significant past medical history was noninsulin‐dependent Type 2 diabetes with a mildly elevated hemoglobin A1c value of 7.7% on the day of admission. The synergism between diabetes and TB is complex. People with diabetes are at greater risk for developing active TB due to immune dysregulation and complex processes, and bidirectional screening has been encouraged in TB endemic countries due to the prevalence of both chronic comorbidities [15, 16]. Studies examining the effect of Type 2 diabetes in patients with TB show increased biochemical dysregulation, variations in liver enzymes, and possibly an intricate relationship with lipids [17, 18]. Elevation in liver enzymes has been suggested to be a cause among providers of holding metformin during antituberculous therapy due to concern for hepatotoxicity, and there is lack of standardization of when to restart making it difficult to tightly control diabetes during this crucial period [17]. Formal immunoglobulin levels, other than IgA which was normal, were not evaluated in this patient, a limitation of the study recognized by the authors. However, the patient did not report a history of recurrent sinopulmonary infections, allergies, or autoimmune diseases in his past medical history, and there was no suspicion for classic immunodeficiency disorders.

The decision was made not to treat for CMV infection. CMV disease is typically treated with ganciclovir/valganciclovir in immunocompromised patients with evidence of tissue invasion, but treatment is less standardized in immunocompetent or mildly immunocompromised patients. The patient’s only abdominal symptom was pain. Ulceration of the ascending colon and narrowing of the ileum seen on imaging and colonoscopy was consistent with colonic tuberculosis without signs of CMV colitis such as edema and diarrhea. The patient did not exhibit symptoms or signs of disseminated CMV with other end‐organ disease such as hepatitis, retinitis, or esophagitis. The patient never exhibited hemodynamic instability or required escalation of care to the ICU during his hospitalization. Given the low‐level CMV viremia, the absence of severe CMV‐attributable symptoms, and strong evidence of disseminated tuberculosis as the unifying diagnosis, CMV reactivation was interpreted as localized reactivation and expected to resolve with treatment of TB. The patient was monitored without anti‐CMV therapy and improved with antituberculosis therapy alone which was confirmed with repeat CT imaging. In summary, immunocompetent patients with TB and CMV gastrointestinal coinfection who do not present with evidence of CMV colitis likely have local CMV reactivation and will improve on antituberculosis therapy alone.

## Author Contributions

Aprilgate Wang: corresponding author, conceptualization, visualization, writing–original draft, and writing–review and editing. Samuel Ji: conception and clinical management. Sarah Semon: conception and clinical management. Sarah Waldman: conception and clinical management. Minh‐Vu H. Nguyen: conception, clinical management, writing–review and editing, and supervision.

## Funding

No funding was received for this study.

## Ethics Statement

Institutional board review was not required for this case report in accordance with institutional policies.

## Consent

The case report does not contain any patient identifiers, and all efforts have been made to ensure patient confidentiality. Multiple unsuccessful attempts were made to reach the patient for verbal consent.

## Conflicts of Interest

The authors declare no conflicts of interest.

## Data Availability

All clinical data supporting the findings of this case report are included within the article.

## References

[1] Tobin E. H. and Khatri A. M. , Abdominal Tuberculosis, 2026, StatPearls Publishing, Treasure Island (FL), https://www.ncbi.nlm.nih.gov/books/NBK556115/. 32310575

[2] Choudhury A. , Dhillon J. , Sekar A. , Gupta P. , Singh H. , and Sharma V. , Differentiating Gastrointestinal Tuberculosis and Crohn′s disease-a Comprehensive Review, BMC Gastroenterology. (2023) 23, no. 1, 10.1186/s12876-023-02887-0. PMC10354965 37468869

[3] Ziebold C. and Pillarisetty L. S. , Congenital Cytomegalovirus Infection, 2026, StatPearls Publishing, Treasure Island (FL). 31082047

[4] Cook C. H. and Trgovcich J. , Cytomegalovirus Reactivation in Critically Ill Immunocompetent Hosts: a Decade of Progress and Remaining Challenges, Antiviral Research. (2011) 90, no. 3, 151–159, 10.1016/j.antiviral.2011.03.179. 21439328 PMC3129598

[5] Nagahashi M. , Aoyagi T. , Yamada A. , Rashid O. M. , Adams B. J. , and Takabe K. , Intestinal Co-infection of Tuberculosis and CMV Can Cause Massive Lower GI Bleeding in a Patient with HIV, Journal Surgery Science. (2013) 1, no. 1, 12–15. PMC4109321 25068146

[6] Boaretto Teixeira Fernandes M. , Nogueira Moisés Cardoso P. A. , Bassani Altoé L. et al., Gastrointestinal CMV Disease and Tuberculosis in an AIDS Patient: Synergistic Interaction Between Opportunistic Coinfections, Case Reports Medical. (2018) 2018, 1–7, 10.1155/2018/8047892. PMC6016156 29991949

[7] Virani Z. , Saldanha N. , Chauhan V. K. et al., POS-088 Simultaneous Cytomegalovirus Colitis and Abdominal Tuberculosis Post Kidney Transplant, Kidney International Reports. (2022) 7, no. 9, 10.1016/j.ekir.2022.07.106.

[8] García-Padilla P. , Contreras K. , Serrano P. P. , Sánchez Leon N. , and Lucero Pantoja O. , Mycobacterium tuberculosis and Cytomegalovirus Colitis in a Renal Transplant Patient: a Case Report, Journal of Investigative Medicine High Impact Case Reports. (2022) 10, 10.1177/23247096221139269. PMC9703518 36433691

[9] Müller J. , Tanner R. , Matsumiya M. et al., Cytomegalovirus Infection is a Risk Factor for Tuberculosis Disease in Infants, JCI Insight. (2019) 4, no. 23, 10.1172/jci.insight.130090. PMC6962026 31697647

[10] Fletcher H. A. , Snowden M. A. , Landry B. et al., T-cell Activation is an Immune Correlate of Risk in BCG Vaccinated Infants, Nature Communications. (2016) 7, 10.1038/ncomms11633. PMC4832066 27068708

[11] Olbrich L. , Stockdale L. , Basu Roy R. et al., Understanding the Interaction Between Cytomegalovirus and Tuberculosis in Children: the Way Forward, PLoS Pathogens. (2021) 17, no. 12, 10.1371/journal.ppat.1010061. PMC8659711 34882748

[12] Imlay H. and Limaye A. P. , Current Understanding of Cytomegalovirus Reactivation in Critical Illness, The Journal of Infectious Diseases. (2020) 221, no. Suppl 1, S94–S102, 10.1093/infdis/jiz638. 32134490 PMC7057786

[13] Limaye A. P. , Kirby K. A. , Rubenfeld G. D. et al., Cytomegalovirus Reactivation in Critically Ill Immunocompetent Patients, JAMA. (2008) 300, no. 4, 413–422, 10.1001/jama.300.4.413. 18647984 PMC2774501

[14] Zhang Z. , Liu X. , Sang L. et al., Cytomegalovirus Reactivation in Immunocompetent Mechanical Ventilation Patients: a Prospective Observational Study, BMC Infectious Diseases. (2021) 21, no. 1, 10.1186/s12879-021-06698-0. PMC8482357 34592936

[15] Krishna S. and Jacob J. J. , Feingold K. R. , Adler R. A. , Ahmed S. F. et al., Diabetes Mellitus and Tuberculosis, Endotext [Internet], 2000.

[16] Boadu A. A. , Yeboah-Manu M. , Osei-Wusu S. , and Yeboah-Manu D. , Tuberculosis and Diabetes Mellitus: the Complexity of the Comorbid Interactions, International Journal of Infectious Diseases. (2024) 146, 10.1016/j.ijid.2024.107140. 38885832

[17] Asare A. B. , Asare P. , Yeboah-Manu M. et al., Patients with Tuberculosis and Diabetes Show Altered Clinical and Biochemical Parameters During anti-TB Treatment, Scientific Reports. (2026) 16, no. 1, 10.1038/s41598-026-36529-8. PMC12923770 41639142

[18] Chidambaram V. , Zhou L. , Ruelas Castillo J. et al., Higher Serum Cholesterol Levels Are Associated with Reduced Systemic Inflammation and Mortality During Tuberculosis Treatment Independent of Body Mass Index, Frontiers in Cardiovascular Medicine. (2021) 8, 10.3389/fcvm.2021.696517. PMC8257940 34239907

